# Supplementary material for: Circulating Endocannabinoids and N-Acylethanolamines in Individuals with Cannabis Use Disorder—Preliminary Findings
Source: Brain Sci. 2023 Sep 27;13(10):1375. doi: 10.3390/brainsci13101375 (PMC10605789; doi:10.3390/brainsci13101375)
Supplement: Supplementary file 1 [file brainsci-13-01375-s001.zip › brainsci-2608033-supplementary.pdf]

Supplementary

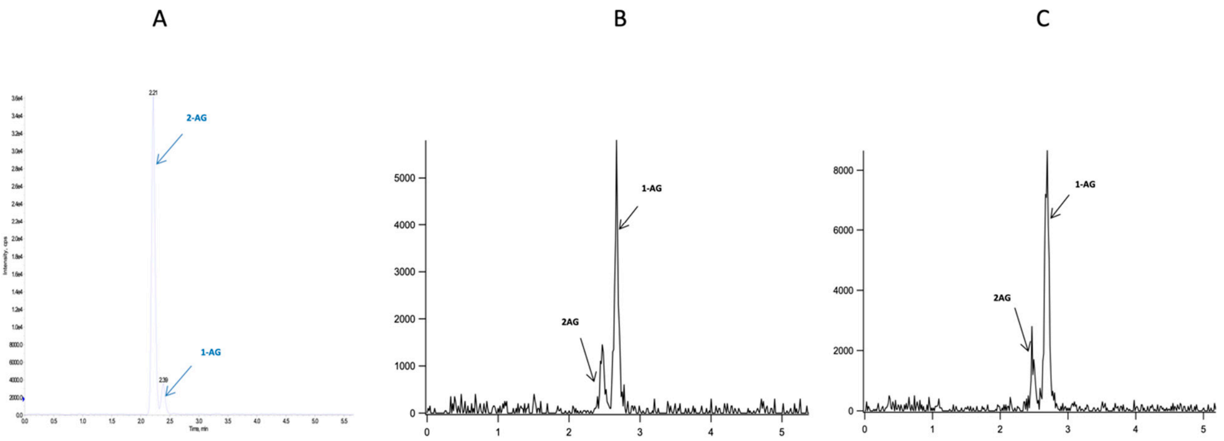

Supplementary Figure S1. Chromatograms of 2-AG in standards and samples. A 2-AG standard, freshly prepared, no extraction process, no storage period; B, 2-AG standard, had extraction process, stored in -80 ^{o}C before LC-MS analysis; C, 2-AG human plasma (e.g. ID FAAH082) had extraction process stored in -80 ^{o}C before LC-MS analysis

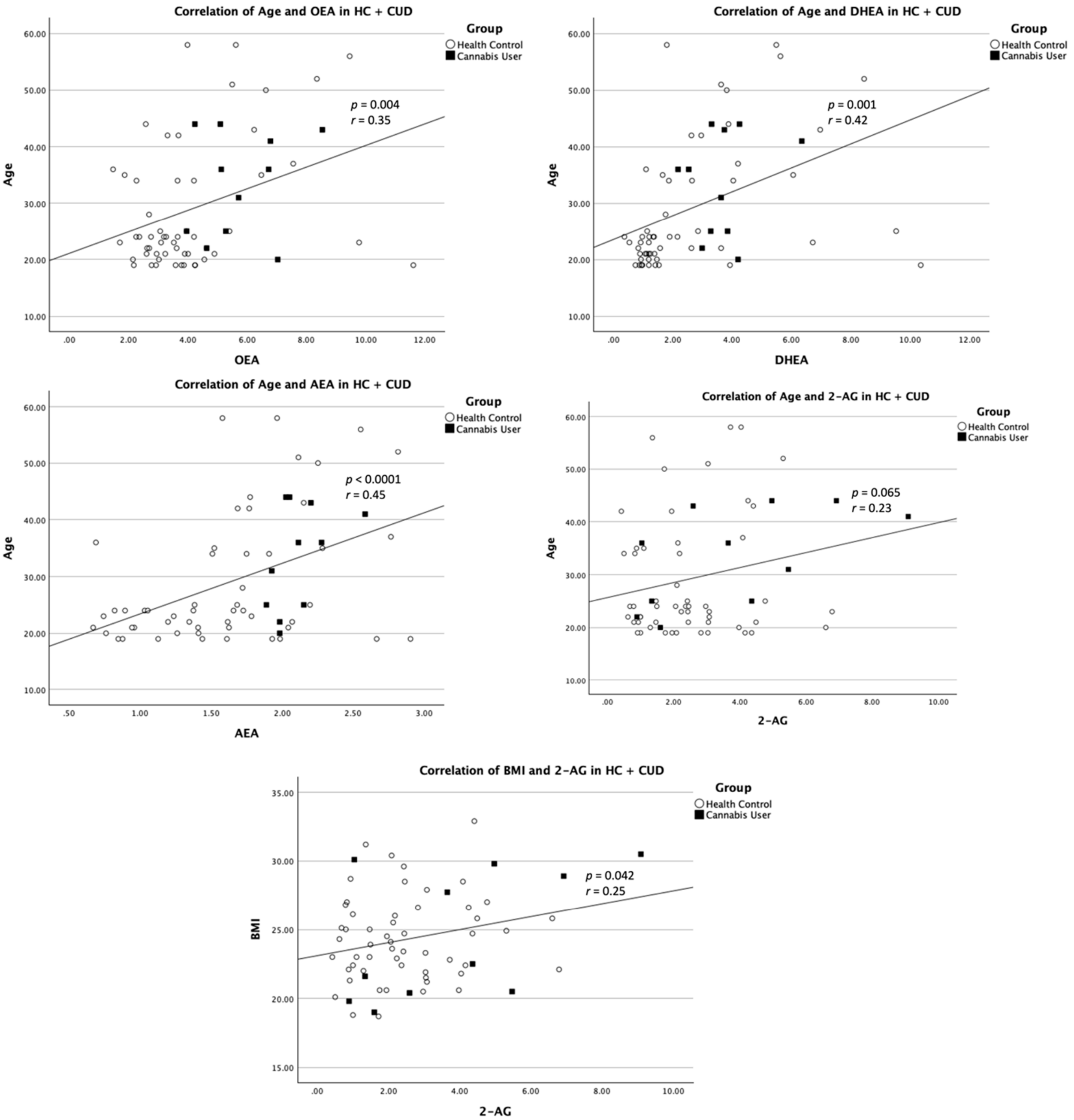

Supplementary Figure S2. Correlation of Body Mass Index (BMI, kg/m2) and Age with Peripheral Endocannabinoids and N-acylethanolamines. Age correlated with anandamide (AEA), N-docosahexaenoylethanolamine (DHEA) and N-oleoylethanolamine (OEA) and marginally with 2-arachidonoylglycerol (2-AG) the whole group Healthy Controls (HC: white square) and Cannabis Users (CU: black squares) combined and in Healthy Controls alone, but not in Cannabis Users alone. BMI only correlated with 2-arachidonoylglycerol (2-AG) in the whole group. BMI did not correlate with any other endocannabinoids and N-acylethanolamines in Cannabis Users, Healthy Controls or combined (whole group) ($r < 0.02$; $p > 0.8$). BMI did not correlate with 2-AG in each group alone ($r < 0.5$; $p > 0.1$).
